# Epileptic Encephalopathy In A Patient With A Novel Variant In The Kv7.2 S_2_ Transmembrane Segment: Clinical, Genetic, and Functional Features

**DOI:** 10.3390/ijms20143382

**Published:** 2019-07-10

**Authors:** Maria Virginia Soldovieri, Paolo Ambrosino, Ilaria Mosca, Francesco Miceli, Cristina Franco, Lorella Maria Teresa Canzoniero, Beth Kline-Fath, Edward C. Cooper, Charu Venkatesan, Maurizio Taglialatela

**Affiliations:** 1 Department of Medicine and Health Science “V. Tiberio”, University of Molise, 86100 Campobasso, Italy; 2 Department of Science and Technology (DST), University of Sannio, 82100 Benevento, Italy; 3 Department of Neuroscience, University of Naples “Federico II”, 80131 Naples, Italy; 4 Department of Radiology, Cincinnati Children’s Hospital Medical Center, University of Cincinnati, Cincinnati, OH 45229, USA; 5 Department of Neurology, Neuroscience and Molecular and Human Genetics, Baylor College of Medicine, Houston, TX 77030, USA; 6 Division of Neurology, Dept. of Pediatrics, Cincinnati Children’s Hospital Medical Center, University of Cincinnati, Cincinnati, OH 45229, USA

**Keywords:** Kv7 channels, voltage sensor, epileptic encephalopathy, retigabine, coupled charge reversal, homology model

## Abstract

Kv7.2 subunits encoded by the *KCNQ2* gene provide a major contribution to the M-current (I_KM_), a voltage-gated K^+^ current crucially involved in the regulation of neuronal excitability. Heterozygous missense variants in Kv7.2 are responsible for epileptic diseases characterized by highly heterogeneous genetic transmission and clinical severity, ranging from autosomal-dominant Benign Familial Neonatal Seizures (BFNS) to sporadic cases of severe epileptic and developmental encephalopathy (DEE). Here, we describe a patient with neonatal onset DEE, carrying a previously undescribed heterozygous *KCNQ2* c.418G > C, p.Glu140Gln (E140Q) variant. Patch-clamp recordings in CHO cells expressing the E140Q mutation reveal dramatic loss of function (LoF) effects. Multistate structural modelling suggested that the E140Q substitution impeded an intrasubunit electrostatic interaction occurring between the E140 side chain in S_2_ and the arginine at position 210 in S_4_ (R210); this interaction is critically involved in stabilizing the activated configuration of the voltage-sensing domain (VSD) of Kv7.2. Functional results from coupled charge reversal or disulfide trapping experiments supported such a hypothesis. Finally, retigabine restored mutation-induced functional changes, reinforcing the rationale for the clinical use of Kv7 activators as personalized therapy for DEE-affected patients carrying Kv7.2 LoF mutations.

## 1. Introduction

The M-current (I_KM_) is a voltage-gated K^+^ current characterized by a low activation threshold, slow activation and deactivation kinetics, and absence of inactivation, which plays a critical role in the regulation of neuronal excitability in the sub-threshold range for action potential generation, thus contributing to network oscillation and synchronization [1]. Subunits encoded by the Kv7.2 (*KCNQ2*) gene provide a major contribution to I_KM_ molecular composition, although additional members of the Kv7 subfamily also play a role in specific regions of the nervous system and at distinct developmental stages [2].

Like other voltage-gated K^+^ channels, functional Kv7 channels are tetramers of subunits, each showing six transmembrane segments (S_1_–S_6_) and cytoplasmic N- and C-termini of variable length; the pore domain is encompassed by the S_5_–S_6_ segments and the intervening linker of each subunit, whereas the transmembrane segments between S_1_ and S_4_ form the voltage-sensing domain (VSD). The C-terminal region of Kv7 subunits contains domains required for homo- or heteromeric subunit assembly [3,4] and for a complex network of mutually interacting molecules, including (but not limited to) phosphatidylinositol 4,5-bisphosphate [5,6], calmodulin [7,8,9], syntaxin [10,11], A-kinase-anchoring proteins and protein kinase C [12], and ankyrin-G [13].

Heterozygous missense variants in Kv7.2 are responsible for epileptic diseases characterized by a highly heterogeneous genetic transmission and clinical severity, ranging from autosomal-dominant Benign Familial Neonatal Seizures (BFNS) to sporadic cases of severe epileptic and developmental encephalopathy (DEE). Several factors likely contribute to the different clinical phenotypes associated with distinct Kv7.2 variants; among those, a major determinant appears to be the mutation-induced functional effects, with more dramatic clinical phenotypes often associated with more severe channel dysfunction in vitro [14,15]. In addition, while most variants reduce K^+^ channel function (loss of function, LoF) [16,17], few cause opposite effects, namely gain-of-function (GoF) effects [18,19]. Understanding the molecular basis underlying the specific functional consequences triggered by Kv7.2 variants, besides shedding light on disease pathophysiology, may also guide patient-tailored therapeutic approaches to be undertaken in patients affected with Kv7.2-related epilepsies.

The pharmacological activation of neuronal Kv7 channels has proven effective to relieve symptoms in several hyperexcitability diseases, such as pain, epilepsy, mania, ADHD, addiction to psychostimulants, and depression. Retigabine is the first-in-class Kv7 channel opener commercialized as an anticonvulsant for several years before being withdrawn from the market in 2017. On the other hand, Kv7 blockers, by potentiating neurotransmitter release, may improve cognitive function in neurodegenerative states, such as Alzheimer disease, although no drug has been approved with this indication [20].

Notably, Kv7 channel activators or inhibitors may ameliorate channel dysfunction and disease progression caused by LoF or GoF variants, respectively, in Kv7.2-related epilepsies. Retigabine has been recently shown to be more effective than phenobarbital in reducing kainic acid-induced seizure burden in vivo in knock-in mice bearing naturally occurring Kv7.2 LoF variants [21], providing a rationale for the use of Kv7 activators in patients carrying these variants [22].

In the present work, we describe the clinical, genetic, functional, and pharmacological properties of a newly identified Kv7.2 mutation (c.418G>C, leading to the E140Q substitution), occurring de novo in a patient affected with neonatal-onset epilepsy and developmental delay. The mutation falls within the VSD domain, but outside the S_3_–S_4_ loop and S4 segment, which is a KCNQ2 DEE hotspot [22,23,24]. E140Q neutralizes a highly conserved negatively charged residue in the S2 transmembrane segment. Functional results revealed that the E140Q variant strongly suppressed Kv7.2 function by markedly decreasing the voltage sensitivity of the opening process, reducing activation kinetics and accelerating deactivation kinetics. Multistate modelling suggested that this effect was due to a mutation-induced loss of an intrasubunit electrostatic interaction occurring between E140 and a positively charged arginine in S4 (R210), involved in stabilizing the activated configuration of Kv7.2 VSD. Functional results from coupled charge reversal and disulfide trapping experiments largely confirmed the occurrence of this interaction, also revealing additional electrostatic interactions contributing to VSD stabilization. Finally, exposure to the Kv7 activator retigabine counteracted the mutation-induced functional effects, reinforcing the rationale for the clinical use of Kv7 activators as personalized therapy for DEE-affected patients carrying Kv7.2 LoF mutations.

## 2. Results and Discussion

### 2.1. Genetic and Clinical Description of the Proband

**Figure 1:**
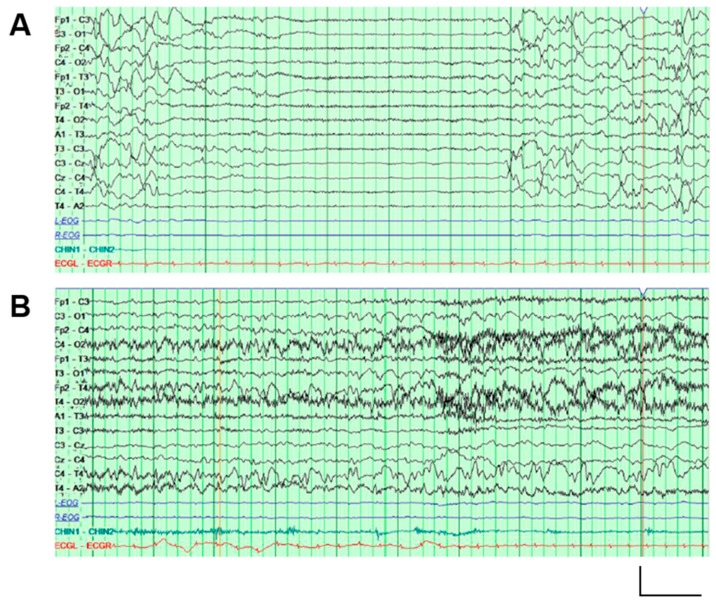
EEG recordings performed on the proband. (**A**) An interictal EEG recording at one day old, showing a representative episode of generalized attenuation lasting 5–6 s. (**B**) An ictal EEG recording (from the same one-day-old study as in panelA), showing evolution of a seizure in the posterior regions of the right hemisphere. Vertical scale bar: 200 μV; horizontal scale bar: 1 s.

The proband is a 29-month-old male with global developmental disability. He was born after unremarkable gestation (39 weeks and 2 days) and delivery. Clinical episodes with tonic arm and leg extension, fist clenching, arching of the back, and dusky color change lasting 10–20 s were first noted at 18 h of life. Continuous video encephalography (EEG) monitoring revealed a background with excessive discontinuity and multifocal epileptiform activity (Figure 1A), and a series of electroclinical seizures arising independently from either the left or right hemisphere (Figure 1B). The seizures initially subsided with phenobarbital therapy, but recurred. Pyridoxine challenge did not produce any favorable change in seizure control, but the addition of levetiracetam and fosphenytoin led to remission. At around 10 days of life, fosphenytoin was changed to topiramate. Seizures again recurred, despite increases in topiramate; therefore, fosphenytoin was resumed, and levetiracetam was discontinued. He was discharged after 41 days on topiramate, fosphenytoin, and phenobarbital. Although pharmacological therapy was established empirically, and genetic diagnosis was only made beyond the neonatal period, the good response to fosphenytoin therapy is in line with the reported effectiveness of sodium channel blockers in patients with Kv7.2 DEE [25].

**Figure 2:**
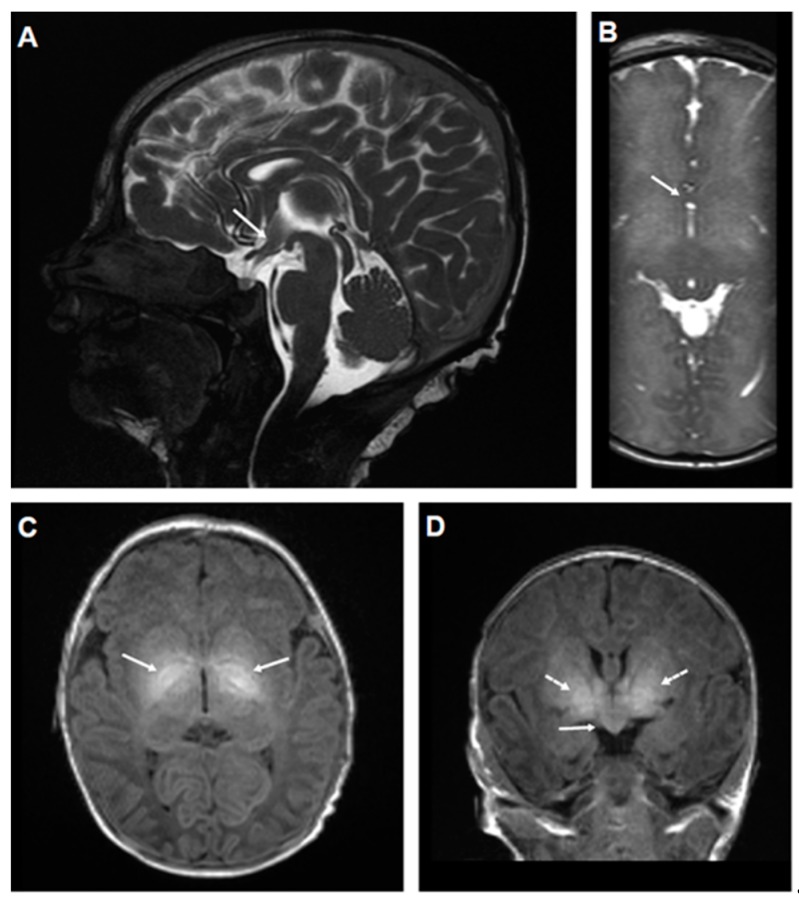
MRI performed on the proband. Neonatal MRI showing septopreoptic fusion abnormality and basal ganglia hyperintensity in a patient with KCNQ2 E140Q: (**A**) Sagittal T2 weighted steady state free procession (SSFP) image demonstrates abnormal tissue (arrow) extending through the anterior recess of the third ventricle in the septopreoptic area; (**B**) axial T2 SSFP image demonstrating interhemispheric fusion of brain parenchyma (arrow), corresponding to the tissue demonstrated in panel (**A**); (**C**) axial T1 image demonstrating abnormal hyperintense signal within the basal ganglia (arrows), especially the globus pallidus; (**D**) coronal T1 image showing both abnormal signal in the basal ganglia as in (**C**) (dotted arrows), and the midline fusion of brain parenchyma in the preoptic area (solid arrow).

Brain MRI performed at 5 and 7 days of age showed a mild fusion anomaly in the spectrum of septopreoptic holoprosencephaly, as well as an abnormal signal within the basal ganglia, most prominently in the globus pallidus (Figure 2). Tests and culture of blood and cerebrospinal fluid (CSF) showed no evidence of infection. Additional testing, including CSF amino acids, neurotransmitters, lactate/pyruvate, serum amino acids, ammonia, glucose, and urine organic acids, was unremarkable.

Phenobarbital and then topiramate were sequentially weaned off by 5 months of age. He was maintained on phenytoin monotherapy until 6 months of age, then transitioned to oxcarbazepine. The patient remained seizure-free, leading to a trial oxcarbazepine discontinuation at 14 months of age, but a seizure occurred after being off medication for approximately 1 month. At last follow-up (age 25 months), he remains seizure-free on oxcarbazepine, 17 mg/kg/day, a relatively low dose.

Despite good seizure control, developmental delays have persisted. He had difficulty with oral feeding in the neonatal intensive care unit and was discharged with a gastrostomy tube; completion of the transition to full oral feeding occurred at 4 months. At 25 months old, he could drink from a straw cup and grasp and eat bite-sized pieces of meat, or soft or softened food, but had difficulty with hard or crunchy food. He first walked at 24 months of age, and remained unsteady and has a wide-based stance at 25 months. Despite receiving ancillary therapies, fine motor skills are delayed, especially those involving bimanual coordination. He makes limited eye contact, grunts, but speaks no words, and appears to have limited receptive language.

Next generation panel sequencing of the patient and both parents identified the Kv7.2 c.418G > C variant (p.Glu140Gln/E140Q) in the proband. This was the only de novo change detected, and was classified by the genetics laboratory as likely pathogenic, using ACMG/AMP criteria [26]. Inherited heterozygous variants in seven other panel genes were detected and classified as of uncertain significance.

Thus, the proband exhibited features highly characteristic of Kv7.2 encephalopathy [22,23,24], namely, onset of focal tonic seizures in the early neonatal period, neonatal encephalopathy without an alternative cause, excessive background discontinuity on neonatal EEG, and subsequent global developmental delay with especially prominent delays in the social and language areas. In addition, the proband presented with two notable abnormalities at neonatal MRI: a bilateral hyperintensity of the lentiform nuclei (Figure 2), which has been previously described as a transient feature associated with Kv7.2 encephalopathy [23,24], and a small hemispheric fusion abnormality in the septopreoptic region, a finding that has never been described in patients affected by Kv7.2 encephalopathy, but as a mild, localized form of holoprosencephaly, and is often associated with mild midline craniofacial anomalies, which were not found in our patient [27]. Although several genes have been implicated in holoprosencephaly, the basis for the rare septopreoptic form has not been elucidated [28,29].

In addition, the network abnormality underlying KCNQ2 patients’ seizures is currently poorly understood. It seems likely that the availability of spontaneously seizing animal models more closely reproducing the genetic abnormality herein presented in one with novel clinical and experimental functional imaging methods to localize the seizure regions and networks involved [30] may fill the current knowledge gap existing between the clinical and molecular studies presented here.

Therefore, since the identified variant was both novel and outside the hotspots where Kv7.2 EE variants are most frequently found [22,23,24] and considering that the patient did not show midline craniofacial anomalies typically detected in holoprosencephaly, in vitro functional characterization of Kv7.2 subunits carrying the E140Q variant was performed to further assess its pathogenic role.

### 2.2. The E140Q Substitution Prompts Loss-of-Function Effects on Kv7.2 Channels

**Figure 3:**
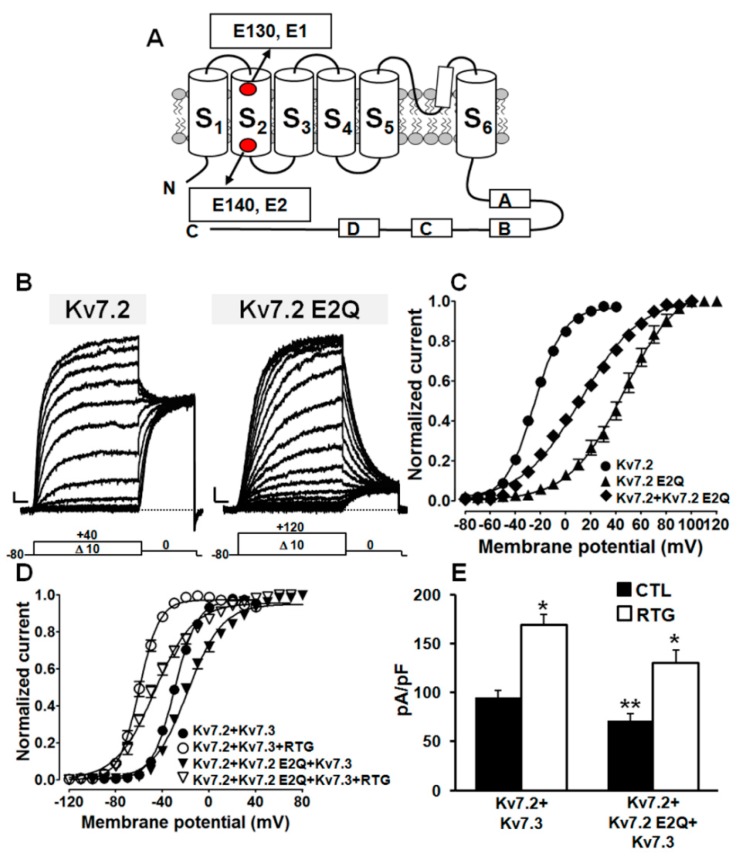
Functional characterization of channels incorporating Kv7.2 E2Q subunits. (**A**) Topology of a single Kv7.2 subunit, showing the localization of the E130 (E1) and E140 (E2) residues; “S_1_” to “S_6_” indicate transmembrane segments. Boxes labelled with “A”, “B”, “C”, and “D” indicate C-terminal α-helical domains. Representative current traces (**B**) and conductance/voltage (G/V) curves obtained (**C**) from CHO cells expressing the indicated channels. The voltage protocol used for these experiments is shown below the traces. Current scale: 100 pA; time scale: 200 ms. G/V curves (**D**) and current densities (**E**) measured for the indicated channels in control conditions (CTL; filled bars) or upon perfusion of 10 μM Retigabine (RTG; empty bars). A single asterisk indicates values showing statistically significant differences (*p* < 0.05) from the respective control; a double asterisk indicates a value showing a statistically significant difference (*p* < 0.05) between Kv7.2+Kv7.3 and Kv7.2+Kv7.2 E2Q+Kv7.3 channels.

Similarly to all other voltage-gated K^+^ channels, functional Kv7 channels are tetramers of randomly assembled identical or compatible subunits [31,32,33]. The Kv7.2 E140Q mutation identified in the proband affects one of the two negatively charged residues located in the S_2_ segment of the VSD (Figure 3A), highly conserved in all five Kv7 members, as well as in other K^+^ channels (Figure S1A), the other being the E130 residue in Kv7.2; therefore, in the present work, we refer to these two residues as E1 for E130 and E2 for E140. To investigate the functional consequences prompted by the Kv7.2 E140Q (E2Q) mutation, wild-type or mutant subunits were heterologously expressed in CHO cells, and the resulting current was recorded by patch-clamp electrophysiology in the whole-cell configuration.

Kv7.2 channels elicited outward K^+^ currents, slowly activating at around −40 mV and reaching a maximal conductance at around 0 mV (Figure 3B,C), as previously described [14,18] (Table 1). By contrast, homomeric Kv7.2 E2Q mutant channels showed a large rightward shift in their voltage dependence of activation, together with a marked decrease in the slope of the conductance/voltage (G/V) curve (as indicated by the *k* factor), and a slight reduction in maximal current size (Table 1). In addition, currents from mutant channels showed significantly slower activation kinetics and faster deactivation kinetics (Figure S1B). By contrast, no difference was measured in reversal potential for K^+^ ions between Kv7.2 and Kv7.2 E2Q channels, indicative of pore selectivity being unaffected by the mutation (Figure S1C). Altogether, these features are suggestive of a mutation-induced loss-of-function effect (LoF).

The Kv7.2 E2Q mutation has been identified de novo on a single Kv7.2 allele; thus, homomeric channels composed of only mutant subunits represent a very small proportion of channels formed in vivo. Furthermore, while most I_KM_ in adult superior cervical ganglion neurons are Kv7.2/Kv7.3 heterotetramers [34], expression of Kv7.2 precedes that of Kv7.3 in both rodent and human brains [35,36,37], and the severity of the Kv7.2 encephalopathy has been correlated to a mutation-induced dysfunction of homomeric Kv7.2 channels, possibly because of their critical role in fine-tuning neuronal connections during development [38]. Therefore, Kv7.2 E140Q subunits were co-expressed both with wild-type Kv7.2 subunits and wild-type Kv7.2 and Kv7.3 subunits.

Cells expressing Kv7.2+Kv7.2 E2Q subunits (cDNA ratio 1:1) displayed K^+^-selective currents showing a significant rightward shift in their voltage dependence of activation, although the magnitude of this effect was smaller than that observed for homomeric Kv7.2 E2Q channels (Figure 3C; Table 1); moreover, when compared to Kv7.2-only expressing cells, a decreased slope of the conductance/voltage curve with no concomitant change in maximal current density was also observed. Qualitatively similar effects on activation gating were also observed when mutant Kv7.2 E2Q subunits were co-expressed with wild-type Kv7.2/Kv7.3 subunits (cDNA ratio: 0.5:0.5:1) (Table 1). These data reveal that the extent of gating impairment is proportional to the number of mutant subunits incorporated, being maximal in homomeric configuration (four mutant subunits; ΔmV~−70 mV), intermediate in channels mostly containing two mutant subunits (Kv7.2+Kv7.2 E2Q subunits; ΔmV~35 mV), and smaller in heteromeric channels with Kv7.2 and Kv7.3 (mostly containing a single mutant subunit; ΔmV~−11 mV). Notably, when compared to Kv7.2/Kv7.3, a ΔmV of approximately 45 mV was also observed when Kv7.2 E2Q subunits were co-expressed with Kv7.3 subunits (cDNA ratio: 1:1) (Table 1).

Based on these functional in vitro results and considering the poor sensitivity of DEE patients to traditional antiepileptic drugs, the effects of the Kv7 activator retigabine (RTG, 10 μM) [20] on the channels incorporating mutant subunits were also assessed. Exposure of cells expressing Kv7.2+Kv7.3 or Kv7.2+Kv7.2 E2Q+Kv7.3 channels to 10 μM RTG similarly shifted current voltage dependence of activation in the hyperpolarizing direction by −30 mV and −29 mV, respectively (Figure 3D), also restoring to wild-type levels the maximum current density in mutant-expressing cells (Figure 3E). In fact, the V_½_s values calculated before or after exposure to 10 μM RTG for Kv7.2+Kv7.3 or Kv7.2+Kv7.2 E2Q+Kv7.3 channels were −29.7 ± 0.4 mV and −59.9 ± 0.4 mV (*n* = 14–22; *p* < 0.05), and −18.4 ± 0.7 mV or −46.6 ± 1.0 mV (*n* = 18–21; *p* < 0.05), respectively.

Altogether, these results indicate that the Kv7.2 E2Q variant, newly found in the proband, reduces channel functional activity, a result consistent with its pathogenic role by a LoF mechanism analogous to that already described for other variants in the VSD of Kv7.2 channels [14]. Notably, the Kv7 activator RTG largely counteracted such mutation-induced functional effects (Figure 3E).

### 2.3. Electrostatic Interactions in Distinct Kv7.2 VSD States Revealed by Homology Modelling

**Figure 4:**
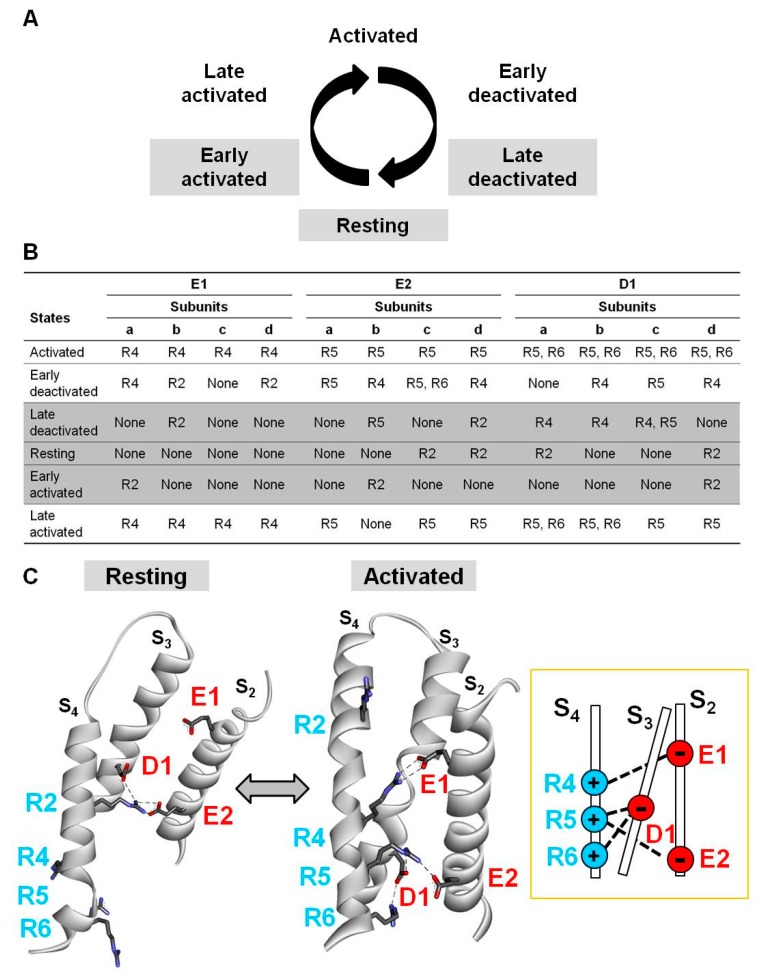
Multistate modelling of Kv7.2 voltage-sensing domain (VSD). (**A**) Schematic representation of six gating states of the VSD. (**B**) Electrostatic interactions identified between E130 (E1), E140 (E2), or D172 (D1) and R201 (R2), R207 (R4), R210 (R5), or R213 (R6) in each gating state for each Kv7.2 subunit (a, b, c, and d). shaded rows correspond to the resting and nearby resting states also highlighted in grey in panel A. (**C**) Homology models of a single Kv7.2 d subunit in resting or activated VSD states. Electrostatic interactions of E1, E2, and D1 residues have been highlighted with dotted lines. The inset on the right indicates a schematic drawing of the VSD interactions occurring in the activated state.

To identify the molecular basis for the observed functional changes prompted by the Kv7.2 E2Q mutation, we used multistate structural modelling. By this technique, structural models of six VSD gating states (activated; early deactivated; late deactivated; resting; early activated; and late activated; Figure 4A) can be built based on long (>200 μs) molecular dynamic simulations of the Kv1.2/Kv2.1 chimera [39] subjected to depolarizing and hyperpolarizing voltages [40], as previously described [18]. The results obtained suggest that the E2 residue, together with two additional negatively charged residues in S_2_ (E130, E1) and S_3_ (D172, D1), is involved in several electrostatic interactions with positively charged residues in S_4_ (R201, R2; R207, R4; R210, R5; R213, R6). Note that the position corresponding to the third R in S_4_ in most other voltage-gated ion channels is occupied by a Q residue in all Kv7 channels (Q204 in Kv7.2) and therefore, no R3 residue will be referred to in the current nomenclature. In particular, each VSD gating state is stabilized by distinct electrostatic interactions, often different in the four subunits, suggesting complex and nonconcerted VSD movements during channel gating [41]. As summarized in Figure 4B for all six gating states in each Kv7.2 subunit, and depicted in Figure 4C for the D subunit only, most of these electrostatic interactions (a total of 11 for E1, 12 for E2, and 17 for D1) occur in the activated VSD state or nearby states (early-deactivated, late-activated), while a much reduced number of interactions involving the same residues (a total of 2 for E1, 5 for E2, and 7 for D1) occur in the resting or nearby states (early-activated, late-deactivated). Moreover, E1, E2, and D1 residues mainly interact with upper (R2) or lower (R4, R5, and R6) S4 charges in the resting and activated states, respectively; this allows rather straightforward predictions on the functional changes, consequent to the neutralization of each S4 R, with a destabilization of the resting state in the case of substitutions involving R2 [18,42,43], and a destabilization of the activated state when the charge at R4, R5, or R6 is neutralized or reversed [14,42,44]. On the other hand, the fact that E1, E2, and D1 are each preferentially involved in interactions stabilizing the activated state is consistent with the present functional data showing that the E2Q variant found in our proband markedly destabilized voltage dependence of channel opening, slowed activation kinetics, and accelerated deactivation kinetics.

### 2.4. Probing E1/R4, E2/R5, D1/R5, and D1/R6 Interactions Using Coupled Charge Reversal Strategy

We next attempted to provide functional evidence for the intrasubunit electrostatic interactions identified by multistate modelling, using coupled charge reversal or disulfide trapping experiments, focusing on those being quantitatively more relevant, namely those involving E1 with R4, E2 with R5, D1 with R5, and D1 with R6. To this aim, electrophysiological experiments were first performed in cells expressing Kv7.2 channels where E1, E2, and D1 residues were substituted with positively charged residues (E130R, E1R; E140R, E2R; D172R, D1R), and R4, R5, and R6 residues were substituted with negatively charged residues (R207E, R4E; R210E, R5E; R210D, R5D; R213D, R6D). Next, coupled charge reversals at each potentially interacting site involving these residues were introduced (E1R/R4E; E2R/R5E; D1R/R5D; D1R/R6D). The occurrence of a specific interaction between mutated residues could be hypothesized when the functional properties of channels formed by these double mutant subunits were different from those of either single mutant.

**Figure 5:**
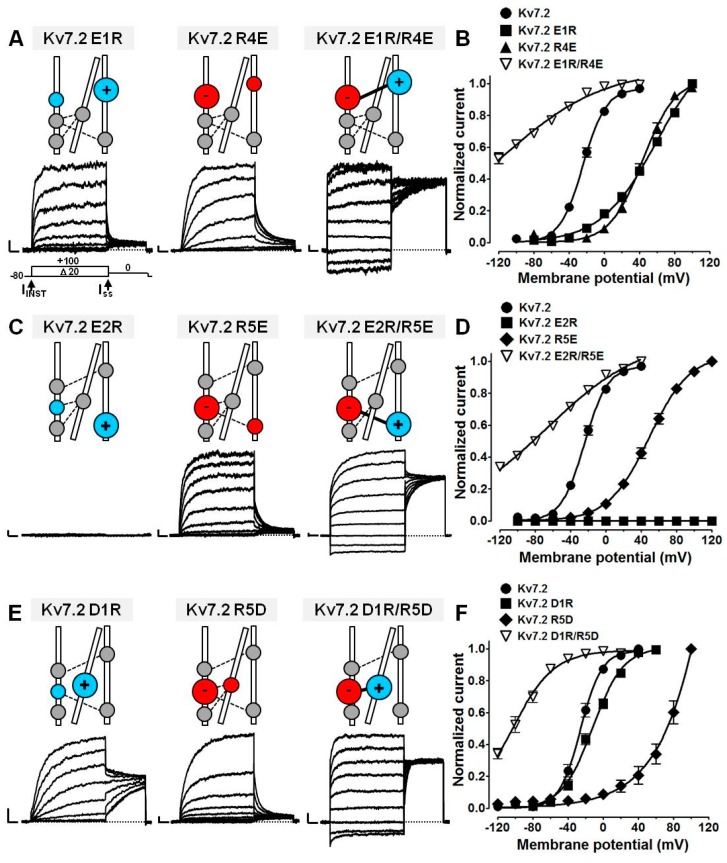
Functional testing of E1/R4, E2/R5, D1/R5 interactions by charge mutagenesis. Representative current traces (**A**,**C**,**E**) and G/V relationships (**B**,**D**,**F**) for the indicated channels expressed in CHO cells. Current scale: 500 pA; time scale: 200 ms. The residues indicated in coloured symbols of larger size in the schematic drawings above panels A, C, and E highlight those where mutations have been engineered. Blue indicates positively charged residues; red indicates negatively charged residues.

Cells expressing Kv7.2 E2R or R6D channels failed to elicit currents above background levels (Figure 5; Table 1), despite mutant subunits being detected at the plasma membrane (Figure S2). All other single-substituted Kv7.2 channels herein investigated were functional, and showed a rightward shift in their activation gating (Figure 5; Table 1), confirming that each residue plays a prevalent role in the stabilization of the Kv7.2 activated state. Consistent with our results is the LoF effect observed in Kv7.1 [45,46] or Kv7.2 [38,42] when the charges at E1, R4, R5, and R6 are neutralized or reversed.

Notably, double-substituted E1R/R4E (Figure 5A,B), E2R/R5E (Figure 5C,D), or D1R/R5D (Figure 5E,F) channels showed opposite functional alterations, with a strong leftward shift in their voltage dependence of activation, generating a significant fraction of time- and voltage-independent currents at resting voltages. In fact, the ratios between the instantaneous (I_INST_) versus the steady-state (I_SS_) currents were 1.0 ± 0.4% (*n* = 10), 79.0 ± 2.6% (*n* = 29), 46.3 ± 2.8% (*n* = 12), and 72.0 ± 4.0% (*n* = 13) for wild-type, E1R/R4E, E2R/R5E, and D1R/R5D Kv7.2 channels, respectively (*p* < 0.05 versus Kv7.2). These currents showed a strong sensitivity to blockade by 3 mM extracellular tetraethylammonium (TEA); the percentage of current inhibition was, in fact, 93.8 ± 1.6% (*n* = 6), 90.2 ± 3.1% (*n* = 3), 87.4 ± 3.3% (*n* = 5), and 93.7 ± 0.8% (*n* = 5) for wild-type, E1R/R4E, E2R/R5E, and D1R/R5D Kv7.2 channels, respectively (*p* > 0.05 versus Kv7.2), strongly arguing in favour of these currents being carried by channels containing Kv7.2 subunits. These results, showing that opposite gating changes were observed between double- and single-substituted mutant channels, argue in favour of an interaction between the two residues in which charges have been swapped. Moreover, the fact that, similarly to the effect of E1 and R4 charges swapping in Kv7.1 [45], activation gating in double-substituted channels was even more negative than that observed in wild-type channels might be explained by the fact that each residue is simultaneously involved in multiple interactions; swapping the charge at one site would therefore introduce a repulsive effect on neighbouring, similarly charged residues, possibly leading to a local amplification of the electrostatic state destabilization and to the “overshooting” effect on channel function. Instead, Kv7.2 D1R/R6D channels were non-functional (Table 1); therefore, the potential occurrence of the electrostatic interaction between D1 and R6 could not be functionally tested.

All interactions predicted by multistate modelling occur within each subunit; instead, charge swapping at the same residues in different channel subunits failed to modify channel gating behaviour when compared to single-substituted channels. In fact, co-expression of mutant subunits, each carrying single charge reversal at the predicted interacting residues in different subunits (E1R + R4E, E2R + R5E, or D1R + R5D), failed to reproduce gating behaviour observed in Kv7.2 channels, in which charge swapping occurred in the same subunits (E1R/R4E, E2R/R5E, or D1R/R5D, respectively) (Table 1). These results therefore confirmed that the functional properties observed depended on intrasubunit, rather than intersubunit, interactions involving the mutated residues. This view is also consistent with the radial position occupied by each VSD in tetrameric Kv7.2 channels, whose reciprocal distance would render highly unlikely the occurrence of intersubunit electrostatic interactions.

### 2.5. Probing E1/R4, E2/R5, D1/R5, and D1/R6 Interactions Using Cysteine Substitutions in Disulfide Trapping Experiments

To provide further support for the hypothesis that the interactions between E1/R4, E2/R5, D1/R5, and D1/R6 residues stabilized the activated VSD configuration, disulfide trapping experiments at the same positions were performed. To this aim, cysteines were first introduced at each of these positions (thus generating E1C, E2C, D1C, as well as R4C, R5C, and R6C mutants), and the functional properties of these single-substituted channels were compared with those of channels carrying double cysteine substitutions at each potentially interacting residues, namely E1C/R4C, E2C/R5C, D1C/R5C, and D1C/R6C Kv7.2 channels. Next, intrasubunit interactions, occurring between E1/R4, E2/R5, D1/R5, and D1/R6 residues, were reversibly probed by exposing these cysteine-substituted channels to reducing (dithiothreitol, DTT) or oxidant (hydrogen peroxide, H_2_O_2_) conditions, thereby cleaving or reforming, respectively, disulfide bridges between thiol groups of newly introduced cysteines.

**Figure 6:**
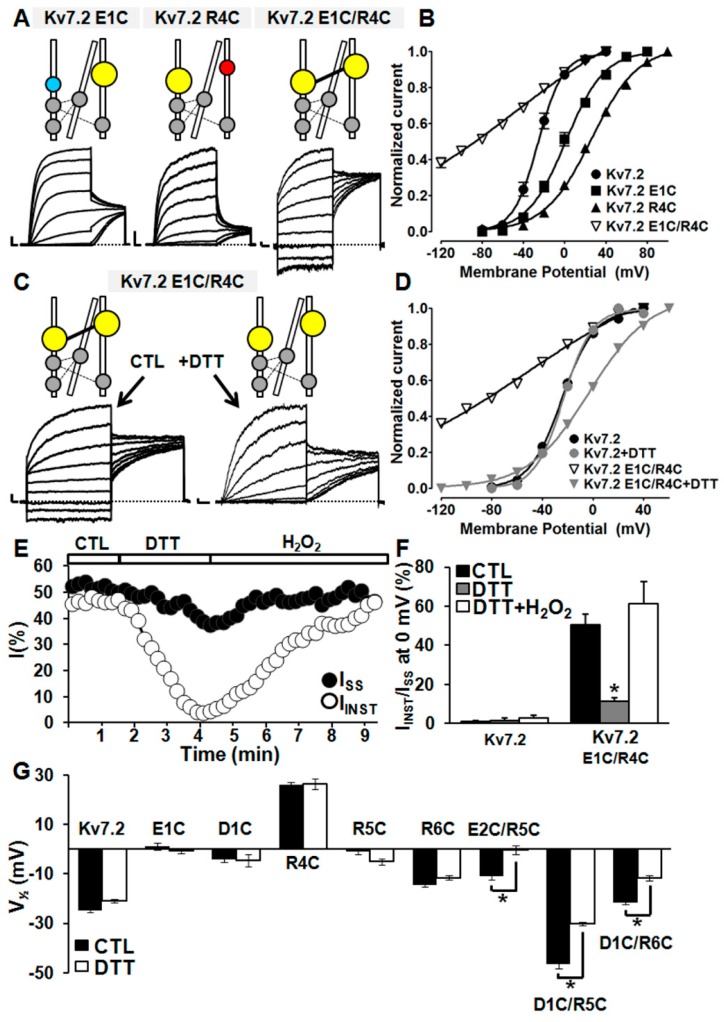
Functional testing of E1/R4, E2/R5, and D1/R5, or D1/R6 interactions by cysteine mutagenesis. Representative current traces (**A**) and G/V curves (**B**) for the indicated channels. Current scale: 500 pA; time scale: 200 ms. Representative traces (**C**) and G/V curves (**D**) in CHO cells expressing Kv7.2 E1C/R4C channels in control solution or upon application of 1 mM dithiothreitol (DTT), as indicated by arrows. (**E**) Time-course of the effects of acute perfusion of DTT (1 mM), followed by H_2_O_2_ (0.5 mM) on instantaneously activated (I_INST_) or time-dependent (I_SS_) currents, expressed by Kv7.2 E1C/R4C channels. (**F**) Quantification of the effects of DTT (1 mM) and H_2_O_2_ (0.5 mM) on cells expressing the indicated channels. The asterisk indicates a value significantly different from that measured in control solution. (**G**) V_½_s of indicated channels in the absence (black bars) or in the presence (white bars) of 1 mM DTT, as indicated. Asterisks indicate significantly different values (*p* < 0.05) versus the values obtained for the same channels in the absence of DTT.

Both E1C and R4C single-substituted Kv7.2 channels, similarly to previously described E1R and R4E, were functional and elicited voltage- and time-dependent outward currents, showing a significant rightward shift in their voltage dependence of activation; by contrast, E1C/R4C channels, similarly to E1R/R4E, prompted opposite functional effects, showing a large fraction of voltage- and time-independent currents (50.7 ± 5.2%; *n* = 13; *p* < 0.05 versus Kv7.2) (Figure 6A,B,F), a result consistent with the stabilization of the Kv7.2 VSD activated configuration when mutations were both simultaneously present. Perfusion of E1C/R4C channels with DTT (1 mM) significantly reduced the I_INST_/I_SS_ ratio, largely restoring the gating behaviour of wild-type Kv7.2 channels (Figure 6C–F). Intriguingly, DTT-induced effects were fully reversed by subsequent perfusion with 0.5 mM H_2_O_2_ (Figure 6E,F). All these described functional changes appeared to be dependent on the presence of both newly introduced cysteines, since no significant effect was prompted by 1 mM DTT on Kv7.2 currents (Figure S3), as previously reported [47], nor on single-substituted E1C or R4C channels (Figure 6G). By contrast, exposure of Kv7.2 channels to 0.5 mM H_2_O_2_ only caused a small increase in maximum current density and a slight leftward shift of about 6 mV in the voltage dependence of activation, with no change in I_INST_/I_SS_ ratio (Figure S3). These results strongly suggest that a DTT- and H_2_O_2_-sensitive interaction between E1C and R4C residues is responsible for the functional changes occurring in double-substituted E1C/R4C channels, arguing in favour of the occurrence of electrostatic interactions between native E1 and R4 residues in Kv7.2 channels.

A similar strategy was used to test the possible occurrence of the E2/R5, D1/R5, and D1/R6 interactions identified by multistate modelling. Like E2R channels, Kv7.2 E2C channels also failed to produce measurable currents, although a significant expression of mutant channel subunits was detected at the plasma membrane (Figure S2). By contrast, D1C and R5C (similarly to D1R and R5E), as well as R6C Kv7.2 channels elicited voltage- and time-dependent outward currents, showing a small but detectable rightward shift of their voltage dependence of activation when compared to wild-type Kv7.2 channels (Figure 6G). Instead, when compared to single-substituted channels, Kv7.2 currents carried by E2C/R5C, D1C/R5C, and D1C/R6C double mutant channels showed an opposite functional behaviour, characterized by a significant leftward shift in their voltage dependence of activation (Figure 6G). Perfusion with 1 mM DTT caused a rightward shift in E2C/R5C, D1C/R5C, and D1C/R6C voltage dependence of activation (Figure 6G). By contrast, subsequent exposure to 0.5 mM H_2_O_2_ largely recovered the functional properties observed before DTT exposure in E2C/R5C, D1C/R5C, and D1C/R6C Kv7.2 channels (data not shown), suggesting the occurrence of specific electrostatic interactions also between E2 with R5, D1 with R5, and D1 with R6.

## 3. Materials and Methods

### 3.1. Genetic Testing

Patient 1 underwent genetic testing as part of clinical care in a CLIA certified laboratory. The test performed was the Pediatric Neurology Region of Interest Trio panel (Claritas Genomics, Cambridge, MA, USA). Next generation sequencing of overlapping gene sets linked to brain malformations (414 genes) and seizures/epilepsy (455 genes) was performed in parallel on proband and parental samples.

### 3.2. Mutagenesis

Plasmids encoding for Kv7.2 subunits carrying a single charge reversion (E1R, E2R, D1R, R4E, R5E, R5D, or R6D) or a single cysteine substitution (E1C, E2C, D1C, R4C, R5C, or R6C) were obtained by Sequence by Overlap Extension (SOE) or PCR Quick-change mutagenesis on a pcDNA3-Kv7.2 construct, as previously reported [48,49]. To obtain the plasmids containing paired substitutions, a BspEI/BsmBI cassette was extracted from Kv7.2 R4E-, Kv7.2 R5E-, Kv7.2 R5D-, Kv7.2 R6D-, Kv7.2 R4C-, Kv7.2 R5C-, or Kv7.2 R6C-encoding plasmids and introduced respectively into Kv7.2 E1R-, Kv7.2 E2R-, Kv7.2-D1R-, Kv7.2 E1C-, Kv7.2 E2C-, or Kv7.2 D1C-encoding plasmids, previously reacted with the same enzymes.

Direct sequencing of the entire coding region for all plasmids was performed to verify the successful incorporation of the desired mutations.

### 3.3. Cell Cultures and Transfections

Wild-type and mutant cDNAs were expressed in Chinese Hamster Ovary (CHO) cells by transient transfection. CHO cells were grown in 100 mm plastic Petri dishes in Dulbecco’s Modified Eagle Medium (D-MEM) containing 10% fetal bovine serum, non-essential amino acids (0.1 mM), penicillin (50 U/mL), and streptomycin (50 μg/mL) in a humidified atmosphere at 37 °C with 5% CO_2_. For electrophysiological experiments, the cells were seeded on glass coverslips (Carolina Biological Supply Company, Burlington, NC, USA) and transfected the next day using Lipofectamine 2000 (LifeTechnologies, Milan, Italy). Total cDNA in the transfection mixture was kept constant at 4 μg. For western blotting experiments, CHO cells were plated on 60 mm plastic Petri dishes at 70% confluence and, the next day, were transiently transfected using Lipofectamine 2000. Total cDNA in the transfection mixture was kept constant at 6 μg. A plasmid encoding for the Enhanced Green Fluorescent Protein (Clontech, Palo Alto, CA, USA) was used as a transfection marker.

### 3.4. Cell Surface Biotinylation and Western Blot

Total or plasma membrane expression of Kv7.2 subunits in CHO cells was investigated by surface biotinylation and western blotting analysis, as described [11]. Channel subunits were identified using mouse monoclonal anti-Kv7.2 primary antibodies (clone N26A/23, dilution 1:1000; Antibodies Inc., Davis, CA, USA), followed by horseradish peroxidase (HRP)-conjugated anti-mouse secondary antibodies (clone NA931V; dilution 1:5.000; GE Healthcare, Little Chalfont, UK). Reactive bands were detected by chemiluminescence (ECL Western Blotting Substrate, Promega Corporation, USA). Images were captured, stored, and analyzed with the Image Lab, version 4.1 analysis software (Bio-Rad Laboratories, Segrate, Italy). An anti-α-tubulin antibody (dilution 1:5000; Sigma, Milan, Italy) was used to check for equal protein loading.

### 3.5. Whole-Cell Electrophysiology

Macroscopic currents from transiently transfected CHO cells were recorded at room temperature (20–22 °C) one day after transfection with an Axopatch 200A amplifier (Molecular Devices, Union City, CA, USA), using the whole-cell configuration of the patch-clamp technique, with glass micropipettes of 3–5 MΩ resistance. The extracellular solution contained (mM): 138 NaCl, 2 CaCl_2_, 5.4 KCl, 1 MgCl_2_, 10 glucose, and 10 HEPES, pH 7.4 with NaOH. The pipette (intracellular) solution contained (mM): 140 KCl, 2 MgCl_2_, 10 EGTA, 10 HEPES, 5 Mg-ATP, pH 7.3-7.4 with KOH. The pCLAMP software (version 10.2; Molecular Devices, Union City, CA, USA) was used for data acquisition and analysis.

Linear cell capacitance (*C*) was determined by integrating the area under the whole-cell capacity transient, evoked by short (5–10 ms) pulses from −80 to −75 mV with the whole-cell capacitance compensation circuit of the Axopatch 200A turned off. All illustrated and analyzed currents were corrected offline for linear capacitance and leakage currents using standard subtraction routines of the Clampfit module of pClamp 10. Current densities (expressed in pA/pF) were calculated as peak K^+^ currents at saturating voltages divided by *C*. Data were acquired at 0.5–2 kHz and filtered at 1–5 kHz with the 4-pole lowpass Bessel filter of the amplifier. No corrections were made for liquid junction potentials. To generate conductance/voltage (G/V) curves, the cells were held at –80 mV and then depolarized for 1.5 s from −80 to +40/+120 mV in +10 mV or +20 mV increments, followed by an isopotential pulse at 0 mV of 800 ms duration. The current values recorded at the beginning of the 0 mV pulse were normalized and expressed as a function of the preceding voltages. The data were fit to a Boltzmann equation of the following form: *y* = *max*/[*1* + *exp*(*V*_1/2_ − *V*)/*k*], where *V* is the test potential, *V*_1/2_ is the half-activation potential, and *k* is the slope factor. To estimate the fraction of instantaneously activated currents, the ratio between the currents measured at the beginning of the depolarization step (I_INST_) and those at the end of the 0 mV depolarization (I_steady-state_, I_SS_) was calculated. Activation kinetics were measured as previously described [14,50]. Briefly, current traces were fit to a single- or a double-exponential function; in the latter case, a single time constant representing the weighted average of the slow and fast components was obtained by using the following equation: τ = (τfA_f_ + τ_s_A_s_)/(A_f_ + A_s_). By contrast, deactivation kinetics were measured, as previously described [14], by fitting to a single exponential function the currents’ tails measured after a +40/+100mV depolarizing step from −120 mV to +20 mV in Δ10 mV incremental steps.

In the experiments with TEA or retigabine (obtained from Valeant Pharmaceuticals, Aliso Viejo, CA, USA) currents were activated either by 3 s voltage ramps from −80 mV to + 80 mV at 0.08 Hz frequency or by the same previously described voltage protocol to assess the G/V. Drug-induced effects were estimated after 1–2 min of drug application.

### 3.6. Multistate Structural Modelling

Three-dimensional models of Kv7.2 subunits were obtained as previously described [18]. Briefly, they were generated by using as templates the coordinates of six different states of Kv1.2/2.1 paddle chimera (PDB accession number 2R9R; 29% of sequence identity with Kv7.2), obtained in molecular dynamics simulations [40]. Modelling of the S_1_–S_4_ VSD in each state was performed with SWISS-MODEL, as described [14]. The models were optimized through all-atom energy minimization by using the GROMOS96 implementation of Swiss-PDBViewer, and analyzed using both the DeepView module of Swiss-PDBViewer (version 4.0.1; http://spdbv.vital-it.ch/) and PyMOL (http://www.pymol.org/).

### 3.7. Statistics

Data are expressed as the mean ± SEM. Statistically significant differences between the data (*p* < 0.05) were evaluated with the Student’s *t*-test or by the ANOVA, when multiple groups were compared.

## 4. Conclusions

The present results suggest that the newly described E140Q (E2Q) variant identified in a proband affected with neonatal-onset DEE caused a drastic depolarizing shift in the opening voltage dependence of Kv7.2 channels, in one with a drastic decrease in activation kinetics and an acceleration in deactivation kinetics. The observed functional changes, suggestive of a strong LoF mechanism, argue in favor of a pathogenic role for the E2Q variant and are consistent with those triggered by other variants responsible for Kv7.2 DEE, including one recently reported in a four-year-old female with neonatal-onset seizures and delayed neurodevelopmental milestones, carrying a de novo variant at the E1 position [38,51]. This patient joins previous case series describing favorable seizure control responses to NaV blockers in individuals bearing such KCNQ2 LoF variants [25,52]. Homology modeling results suggested that the E2 residue mutated in our proband is involved in a complex network of electrostatic interactions controlling VSD stability at distinct gating states, exerting a prevalent role in stabilizing the activated VSD configuration by a specific interaction with the R210 residue in S_4_. Thus, its neutralization by the E2Q mutation would preferentially stabilize the VSD resting state, a result consistent with the described functional data. Finally, the ability of retigabine to counteract mutation-induced functional effects reinforces the rationale for the use of Kv7 activators in the management of DEE-affected patients carrying Kv7.2 LoF mutations.

## Figures and Tables

**Table 1:** Biophysical properties of currents carried by Kv7 channels.

Channel	*n*	Current Density (pA/pF) ^#^	*V*_1/2_ (mV)	*k* (mV/*e*-Fold)
**Kv7.2**	44–108	33.2 ± 2.0 (0 mV)	−25.0 ± 0.4	11.7 ± 0.3
**Kv7.2 E2Q**	12	19.6 ± 4.5 (+80 mV) *	46.1 ± 2.2 *	21.8 ± 1.3 *
**Kv7.2 E2Q+Kv7.2**	13	39.8 ± 4.0 (+80 mV)	12.4 ± 1.0 *	24.0 ± 0.7 *
**Kv7.3**	28	12.8 ± 1.2 (0 mV)	−38.6 ± 0.4	6.3 ± 0.4
**Kv7.2 E2Q+Kv7.3**	18	97.2 ± 7.8 (+80 mV) **	14.2 ± 1.0 **	22.0 ± 0.7 **
**Kv7.2+Kv7.3**	25	165.2 ± 8.8 (0 mV)	−29.7 ± 0.4	9.8 ± 0.3
**Kv7.2+Kv7.2 E2Q+Kv7.3**	21	119.8 ± 11.7 (+40 mV) **	−18.4 ± 0.7 **	14.8 ± 0.6 **
**Kv7.2 E1R**	7	29.0 ± 4.1 (+100 mV)	59.5 ± 3.4 *	31.7 ± 1.5 *
**Kv7.2 E2R**	23	0.8 ± 0.1 (0 mV) *	-	-
**Kv7.2 D1R**	12–14	13.2 ± 2.2 (+40 mV) *	−11.9 ± 1.5 *	17.5 ± 1.2 *
**Kv7.2 R4E**	12–13	31.8 ± 6.5 (+100 mV)	43.8 ± 1.9 *	18.7 ± 1.4 *
**Kv7.2 R5E**	16	73.7 ± 7.8 (+120 mV) *	49.1 ± 1.7 *	23.1 ± 1.2 *
**Kv7.2 R5D**	8	26.9 ± 7.1 (+100mV)	≈200 *	39.0 ± 7.5 *
**Kv7.2 R6D**	7	0.2 ± 0.2 (0 mV) *	-	-
**Kv7.2 E1R/R4E**	27–30	62.2 ± 6.8 (0 mV) *	-	-
**Kv7.2 E1R+Kv7.2 R4E**	5	56.3 ± 3.4 (+100 mV) *	37.7 ± 1.8 *	22.8 ± 1.2 *
**Kv7.2 E2R/R5E**	13	30.4 ± 5.1 (0 mV)	-	-
**Kv7.2 E2R+Kv7.2 R5E**	5–6	50.0 ± 6.2 (+120 mV) *	53.7 ± 1.7 *	24.1 ± 1.1 *
**Kv7.2 D1R/R5D**	16	54.3 ± 11.7 (+0 mV) *	-	-
**Kv7.2 D1R+Kv7.2 R5D**	9–13	30.2 ± 4.4 (+100 mV)	51.9 ± 5.2 *	34.4 ± 2.4 *
**Kv7.2 D1R/R6D**	9	0.7 ± 0.2 (0 mV) *	-	-

* *p* < 0.05 versus Kv7.2; ** *p* < 0.05 versus Kv7.2+Kv7.3; ^#^ Membrane voltages used to assess current densities are indicated in brackets.

## Supplementary Materials

Supplementary materials can be found at https://www.mdpi.com/1422-0067/20/14/3382/s1.
