# Supplementary material for: Epileptic Encephalopathy In A Patient With A Novel Variant In The Kv7.2 S2 Transmembrane Segment: Clinical, Genetic, and Functional Features
Source: Int J Mol Sci. 2019 Jul 10;20(14):3382. doi: 10.3390/ijms20143382 (PMC6678645; doi:10.3390/ijms20143382)
Supplement: Supplementary file 1 [file ijms-20-03382-s001.pdf]

Suppl. Fig. 1

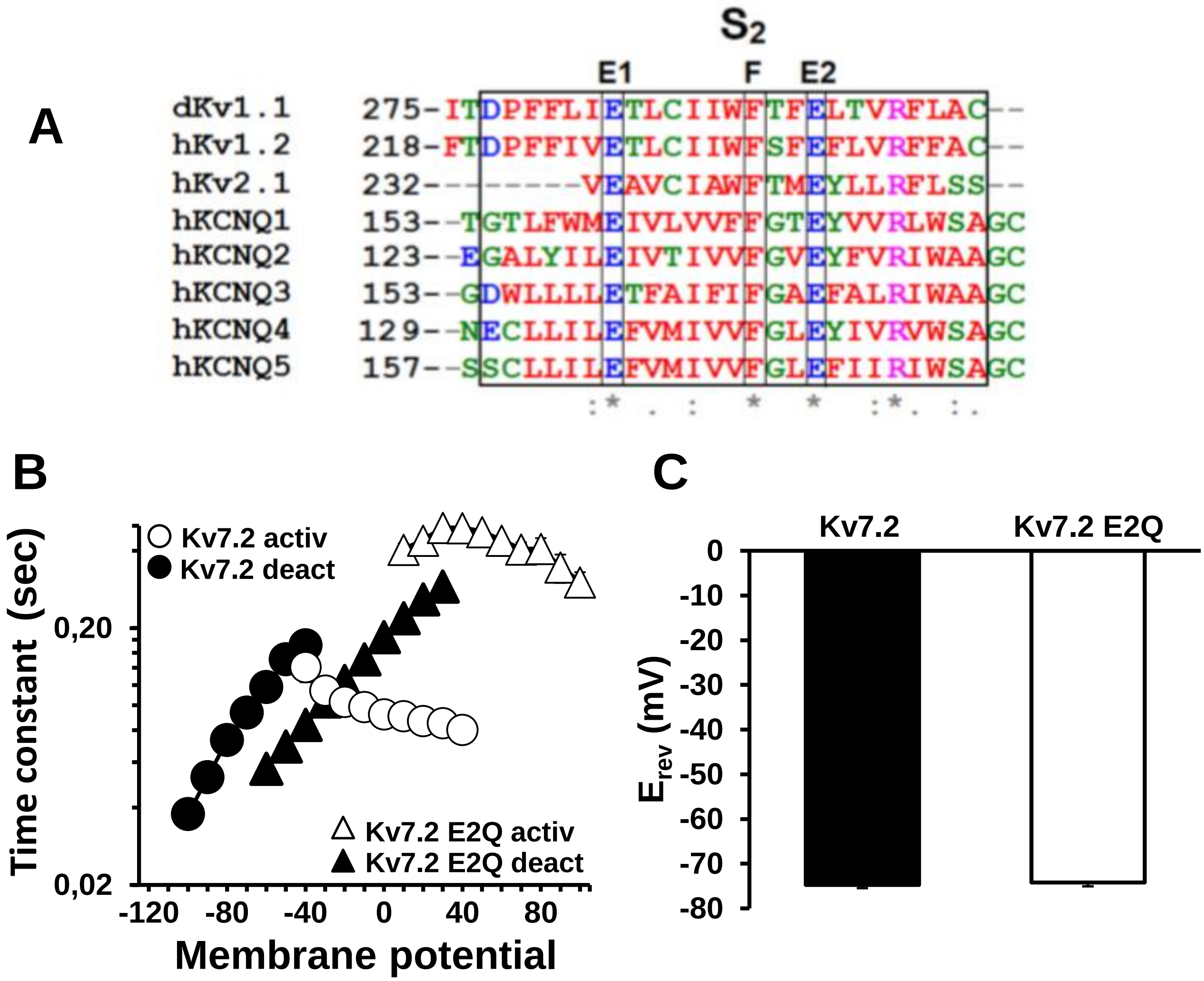

Suppl. Figure 1. A, Partial alignment of S_2 segments of the indicated K^+ channels. «E1» and «E2» indicate the two highly-conserved negatively-charged residues corresponding to E130 and E140 in Kv7.2. B, Quantification of time-constants of activation (n=16) and deactivation (n=11-13) kinetics for Kv7.2 or Kv7.2 E2Q channels, as indicated. C, Quantification of the reversion potentials measured for Kv7.2 or Kv7.2 E2Q channels (n=11-13), as indicated.

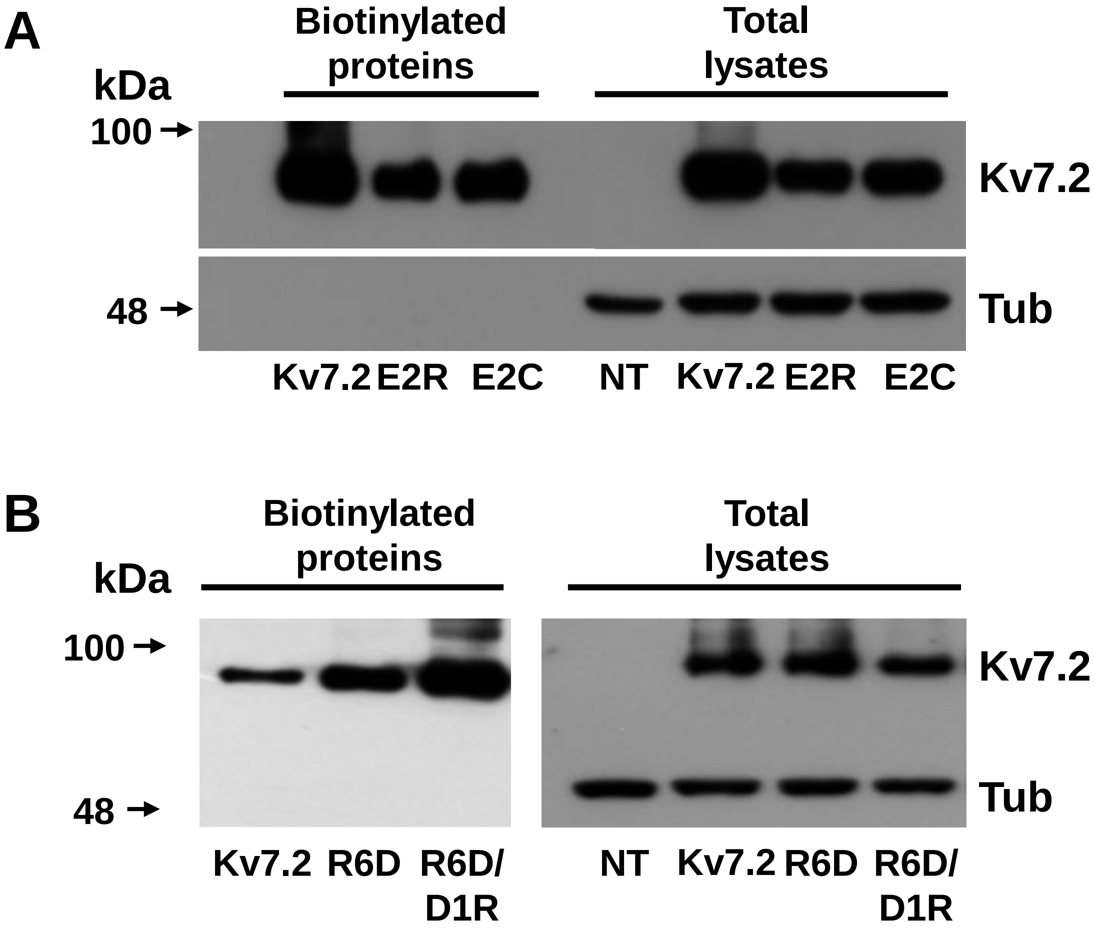

Suppl. Figure 2. Membrane expression of wild-type and mutant Kv7.2 subunits. A,B, Western blot analysis of proteins from total lysates (right) or sptreptavidin-purified biotinylated plasma membrane fractions (left) from untransfected CHO cells (NT) or from CHO cells expressing wild-type or mutant Kv7.2 subunits, as indicated. In each panel, the higher and lower blots were probed with anti-Kv7.2 or anti-α-tubulin antibodies, as indicated. The arrows and the numbers on the left of panels indicate the molecular mass of the weight markers. Data are rappresentative of were replicated at least three experiments.

Suppl. Fig. 3

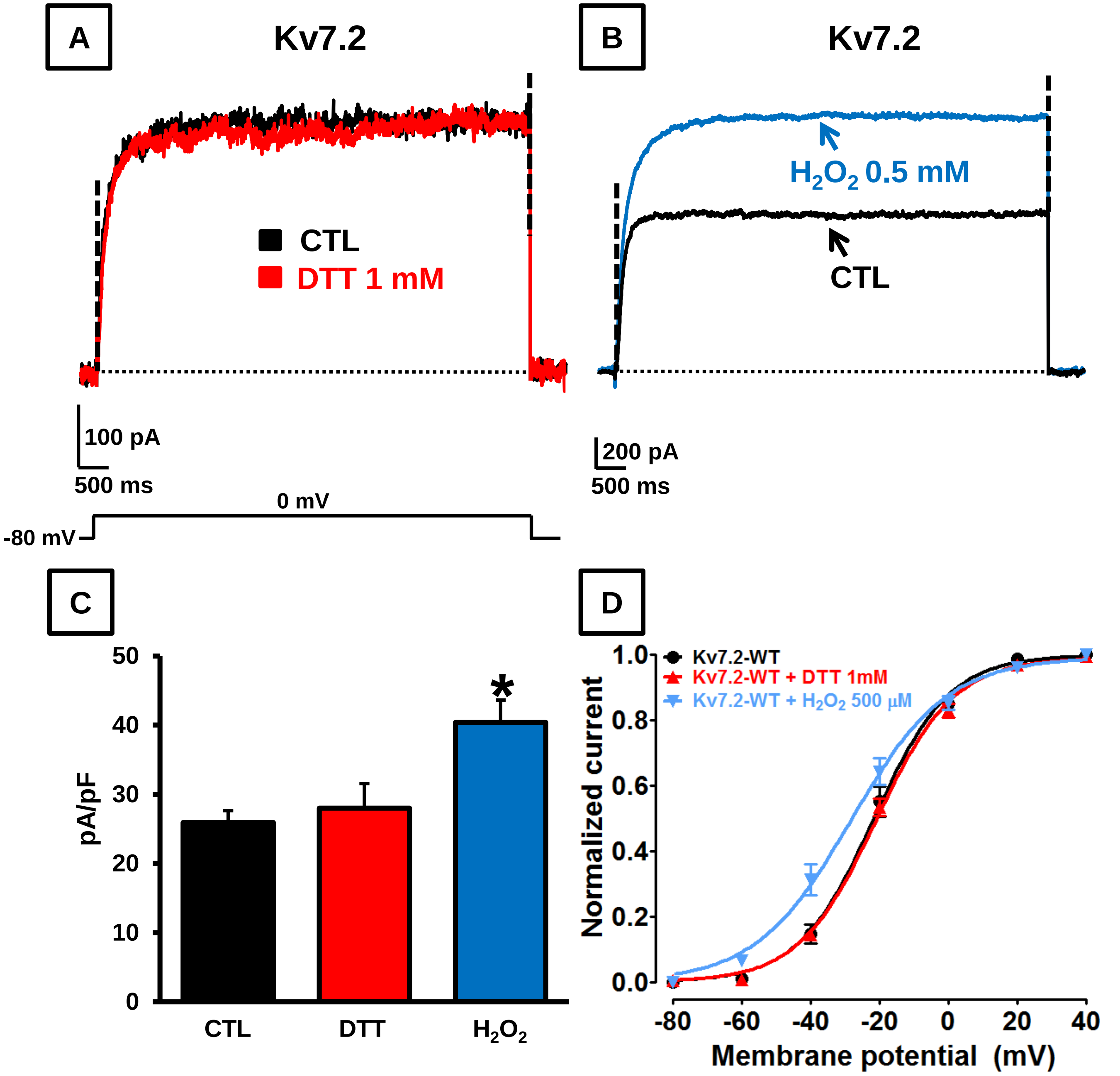

Suppl. Figure 3. Effect of DTT or H_2O_2 on Kv7.2 channels. A,B) Representative traces of Kv7.2 currents measured in response to the indicated voltage protocol in control conditions (black traces) or in the presence of 1 mM DTT (A, red trace) or 0.5 mM H_2O_2 (B, blue trace). C, D) Pooled analysis of the effect of 1 mM DTT or 0.5 mM H_2O_2 on current densities (C) or activation gating (D).
